# Problem-based learning using patient-simulated videos showing daily life for a comprehensive clinical approach

**DOI:** 10.5116/ijme.589f.6ef0

**Published:** 2017-02-27

**Authors:** Akiko Ikegami, Yoshiyuki Ohira, Takanori Uehara, Kazutaka Noda, Shingo Suzuki, Kiyoshi Shikino, Hideki Kajiwara, Takeshi Kondo, Yusuke Hirota, Masatomi Ikusaka

**Affiliations:** 1 Department of General Medicine, Chiba University Hospital, Chiba, Japan

**Keywords:** Problem-based learning, PBL, patient-simulated video, biopsychosocial

## Abstract

**Objectives:**

We examined whether problem-based learning tutorials using patient-simulated videos showing daily life are more practical for clinical learning, compared with traditional paper-based problem-based learning, for the consideration rate of psychosocial issues and the recall rate for experienced learning.

**Methods:**

Twenty-two groups with 120 fifth-year students were each assigned paper-based problem-based learning and video-based problem-based learning using patient-simulated videos. We compared target achievement rates in questionnaires using the Wilcoxon signed-rank test and discussion contents diversity using the Mann-Whitney U test. A follow-up survey used a chi-square test to measure students’ recall of cases in three categories: video, paper, and non-experienced.

**Results:**

Video-based problem-based learning displayed significantly higher achievement rates for imagining authentic patients (p=0.001), incorporating a comprehensive approach including psychosocial aspects (p<0.001), and satisfaction with sessions (p=0.001). No significant differences existed in the discussion contents diversity regarding the International Classification of Primary Care Second Edition codes and chapter types or in the rate of psychological codes. In a follow-up survey comparing video and paper groups to non-experienced groups, the rates were higher for video (χ^2^=24.319, p<0.001) and paper (χ^2^=11.134, p=0.001). Although the video rate tended to be higher than the paper rate, no significant difference was found between the two.

**Conclusions:**

Patient-simulated videos showing daily life facilitate imagining true patients and support a comprehensive approach that fosters better memory. The clinical patient-simulated video method is more practical and clinical problem-based tutorials can be implemented if we create patient-simulated videos for each symptom as teaching materials.

## Introduction

The problem-based learning (PBL) tutorial is a case-based learning method for knowledge acquisition and the development of self-learning abilities that can be used in clinical practice.^1^^,^^2^ As an effective technique for acquiring practical knowledge to connect lectures with clinical practice, PBL has been used in pre-graduation clinical education at many schools of medicine. However, PBL tutorials are often implemented in curricula divided by field. Therefore, the problems extracted from the assigned scenarios are focused on symptoms in that field. There is a concern that discussions may not include psychosocial aspects so that in actual medical examinations, a broad-field approach is required that includes the patient’s psychosocial profile together with considerations of the biological profile.^3^^,^^4^ Therefore, the consideration of biopsychosocial aspects in PBL tutorials is an essential part of the preparation for actual clinical practice.

PBL tutorials have primarily used assignments with paper-based scenarios. However, authentic cases are desirable for improving educational effects,^2^^,^^5^ and in recent years methods with a sense of reality are being considered when using simulated patients (SP) and videos.^5^^-^^7^ On paper, the main information displayed is symptom-related explanations; nonverbal information that is essential in actual clinical practice is excluded. Video enables free thinking by the learner and also makes it possible to directly convey emotions and nonverbal information through visual and auditory information, which makes for the easy imagining of actual cases. There is a cost in producing patient-simulated videos, but these videos offer the benefit of not requiring costs for each PBL, as SP do. Additionally, these videos are available without considering the circumstances of the SP. The research reported here used high-quality patient-simulated videos that were broadcasted on television after obtaining permission from the production company; there were, therefore, no production costs. 

In a past report comparing video- and paper-based PBL, video-based PBL tutorials were preferred by students and tutors and were regarded as offering better learning effects8-10 and memory retention8). The usefulness of videos is controversial; some reports indicate that more students prefer paper-based PBL tutorials^11^^,^^12^ and that videos might hinder students’ clinical reasoning.^12^ Videos used in past reports were limited to scenes of medical interviews and examinations in examining rooms, and so one can surmise that it is difficult to imagine a patient’s daily life, including its social aspects.

The aim of this study is to examine whether the PBL tutorial using patient-simulated videos showing daily lives offers more practical clinical learning, compared with traditional paper-based PBL, about the consideration rate for psychosocial issues and the retention rate for experienced learning.

## Methods

### Study design and participants

A cross-sectional survey was conducted at the Chiba University Hospital’s Department of General Medicine (hereafter referred to as “our department”) in Japan.

The PBL tutorials were conducted from February 2014 to January 2015 as part of clinical clerkship at our department. The participants were all 120 fifth-year students in Chiba University, School of Medicine in 2014. They were randomly divided into 22 groups of five to six students each for clinical clerkship rotations. During the two-week learning period in our department, 22 groups received PBL tutorials of two cases, one video and one paper.

The 120 students were then gathered in a lecture room at the University in July 2015 for a follow-up survey. There were some differences in the time periods between the PBL tutorials and follow-up survey, but the survey was given to all students at the same time so that a high responses rate was received at a uniform place and time.

The tutors were all seven teachers affiliated with our department, and one tutor was assigned to each group. Before the PBL tutorials, the teachers were received lecture and given materials about the PBL tutorials and the role of the tutors. A tutor meeting was held after the completion of all PBL tutorials in February 2015.

This research was conducted with the consent of the participating students and tutors after we received permission from Chiba University, Graduate School of Medicine Ethical Review Committee.

### Data-collection methods and procedure

#### PBL tutorials

**Figure 1:**
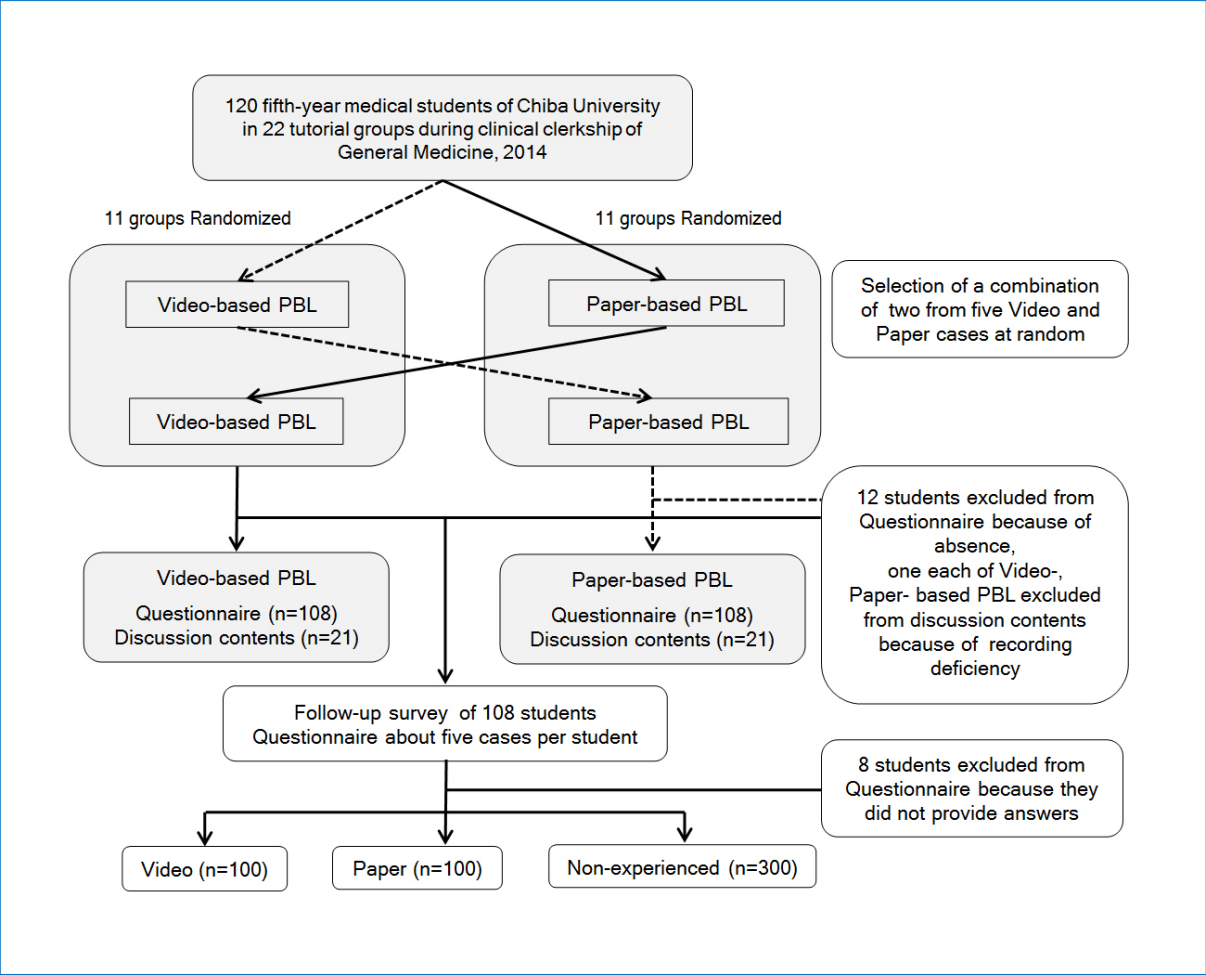
Flow diagram of study design

In the PBL tutorials, we examined the target achievement rates and the discussion contents diversity. During the two-week learning period in our department, the participating students received two PBL tutorials, one each week. The 22 groups were randomly divided in two. The first group was given a PBL tutorial using a simulated video (video-based PBL) in the first week followed by a traditional PBL tutorial in the second week that used a paper-based scenario assignment (paper-based PBL). The second group was given a paper-based PBL in the first week and a video-based PBL in the second week (see Figure 1). In each PBL tutorials, we examined the target achievement rates and discussion contents diversity and compared between video-based PBL and paper-based PBL.

For both PBL, each case was divided in two sessions, for a total of four times, with each session lasting two hours. In the first session, medical history and physical findings were presented, and laboratory findings, imaging findings, and treatment plans were presented in the second session. Digital voice recorder recorded all tutorial sessions.

We randomly chose a combination of two cases from a total of five by using the envelope method. Five cases were implemented: Fitz-Hugh-Curtis syndrome, panic disorder, subacute combined degeneration of spinal cord, primary amyloidosis, and sleep apnea syndrome. A patient-simulated video and paper-based scenario assignment were prepared for each case. These cases were experienced in our department. The patient-simulated videos were produced for a television program on the theme of case investigations and were used for this research with the permission of the production company. The patient-simulated videos included medical history information. In addition to medical interview scenes in examining rooms, they also showed the symptoms displayed in the patient’s home or place of work, in the patient’s daily life, and so on. These videos were simulated by professional actors under medical supervision. The patient-simulated video was shown on a screen, projected from a computer so that the students could view them in detail. The paper-based scenarios were derived from the patient-simulated videos, which were produced by the teachers serving as tutors. These scenarios were distributed to the students. The patient-simulated videos and paper-based scenarios had different medical history information; physical findings, laboratory findings, imaging findings, and treatment plans were distributed as paper materials for both.

### Target achievement rate of PBL tutorials

After the completion of the second PBL tutorial session of each week, the questionnaires were given to each participating students. The students were given identification numbers for anonymity and to correspond the two results, video and paper.

Data was collected using a self-administered five-point Likert scale questionnaire. The questionnaire contents were eight items, included achievement rate evaluations for the five targets of the university’s PBL tutorials (see Table 1, No.1-5). In addition, three items, “Imagining the authentic patient” and “Incorporating a comprehensive approach including psychosocial aspects,” and “Satisfaction with the session” were surveyed (see Table 1, No. 6-8). These contents were determined after focus-group discussion by the author and coauthors on the validity of the contents. Among them, the five targets of the university’s PBL tutorials were determined by faculty members belonging to the medical education department of our university. We verified the reliability of the results using Cronbach’s alpha, which is an index of internal consistency. The scale for the target achievement ratings was based on 1=poor, 2=not very good, 3=neutral, 4=somewhat good, 5=very good. The scale for satisfaction ratings was based on 1=very dissatisfied, 2=dissatisfied, 3=neither satisfied nor dissatisfied, 4=satisfied, 5=very satisfied.

A comparative survey was also given to the students on completion of the two weeks of PBL tutorials to see whether they preferred video- or paper-based PBL. The scale for preference was ratings based on strongly prefer video, prefer video, neutral, prefer paper, strongly prefer paper; the participants were also asked to write the reasons for their preference freely.

The agreement was received from all 120 participating students. A total of 108 (90.0%) were the participants in the questionnaire analysis; this figure excludes 12 who were absent from one or more PBL tutorials.

### Discussion contents diversity of PBL tutorials

Data was collected extracting the differential disease names mentioned in the discussions of each PBL tutorials. One researcher extracted the disease names from audio recordings made with a digital voice recorder and coded them using the disease code (component 7) in the International Classification of Primary Care Second Edition (ICPC-2).13,14 After this, the number of codes, the number of chapter types and the rate of psychology codes were evaluated. The ICPC-2 contains 17 chapters. Chapters are based on body systems with additional chapters for psychological problems and social problems: for example, B is blood and P is psychological. The rate of psychology codes was calculated from the number of psychology codes / the number of all codes × 100(%). The difference between the video- and paper-based PBL was the medical history information presented in the first session; for this reason, only the first sessions for each PBL tutorial were used as targets of the analysis. Two PBL tutorials in which there were recording deficiencies (one video-based PBL and one paper-based PBL) were excluded from the evaluation of the discussion contents; as a result of this, 42 of the 44 PBL tutorials were analyzed (95.5%).

At the tutor meeting, a focus group discussion was held with all tutors of the differences between video- and paper-based PBL.

**Table 1:** Fifth-year medical students’ target achievement rates, based on the 5-point Likert scale, about video- and paper-based PBL, Chiba University Hospital in 2014 (N=108)

Questionnaire (5-point Likert scale)	PBL	25^th*^	Median	75^th**^	p value
1. Structure of knowledge for use in clinical contexts^†^	Video	4	4	5	0.365
	Paper	4	4	5	
2. Development of an effective clinical reasoning process^†^	Video	4	4	5	0.042
	Paper	4	4	5	
3. Development of effective self-directed learning skills^†^	Video	4	4	4	0.78
	Paper	4	4	4	
4. Provision of encouragement and motivation for learning^†^	Video	4	5	5	0.416
	Paper	4	4	5	
5. Development of team skills^†^	Video	4	4	5	0.506
	Paper	4	4	4	
6. Imagining the authentic patient^†^	Video	4	4	5	0.001
	Paper	3	4	4	
7. Incorporating a comprehensive approach including psychosocial aspects^†^	Video	4	4	4	<0.001
	Paper	3	4	4	
8. Satisfaction with the session^‡^	Video	4	5	5	0.001
	Paper	4	4	5	

*25th percentile; **75th percentile; †Scale ratings: 1=poor, 2=not very good, 3=neutral, 4=somewhat good, 5=very good; ‡Scale ratings:1=very dissatisfied, 2=dissatisfied, 3=neither satisfied nor dissatisfied, 4=satisfied, 5=very satisfied.

### Follow-up survey

In the follow-up survey, we examined the memory retention rates of PBL tutorial cases. Data was collected using a self-administered survey form presenting the diseases from the five cases discussed above for each participating students. A time limit of five minutes was established, and the students wrote the symptoms they recalled, without a limit on the number. The survey forms and identification numbers of PBL tutorials were collated. The cases experienced in the video-based and paper-based PBL were identified as either “video” or “paper” cases, and cases that were not experienced in either type of PBL were identified as “non-experienced” cases. The five responses from each student were sorted into three groups, with one question for video, one question for paper, and three questions for non-experienced (see Figure 1). If the symptoms in the response included the chief complaint from the PBL tutorial case, it was determined that the student recalled the case. It is possible that the period from the conclusion of the PBL tutorials until the follow-up survey could influence memory retention. Thus, the responses were evaluated for the video and paper groups taking into account the relationship between presence and absence of recall and the time until the follow-up survey. Responses were received from 100 of the 108 participating students (a response rate of 92.6%), excluding 12 students excluded in target achievement rates evaluation.

**Table 2:** Evaluations of the discussion contents diversity for video- and paper-based PBL, Chiba University Hospital in 2014 (N=42)

ICPC-2^*^	PBL	25^th*^^*^	Median	75^th†^	p value
Number of codes	Video	15.5	19	23.5	0.641
	Paper	16	21	23	
Number of chapter types^‡^	Video	7	7	9	0.247
	Paper	7	8	9	
Rate of psychological codes (%)^¶^	Video	1.4	6.7	11.6	0.071
	Paper	5.2	12.5	15.5	

*ICPC-2 International Classification of Primary Care Second Edition; **25th percentile; †75th percentile; ‡The ICPC-2 contains 17 chapters. Chapters are based on body systems with an additional chapter for psychological problems and one for social problems: for example, B is blood and P is psychological; ¶The rate of the psychology codes = (the number of psychology codes / the number of all codes) ×100(%).

### Data analysis

The data were analyzed on SPSS, version 22.0. The questionnaire of target achievement rates and evaluations of discussion contents diversity were compared for video-based and paper-based PBL (see Figure 1). A Wilcoxon signed-rank test was used to compare the target achievement rates for video-based PBL and paper-based PBL. The discussion contents diversity evaluated according to the number of ICPC-2 codes, the number of ICPC-2 chapter types, and the rate of psychological disease (P) codes, were compared by a Mann-Whitney U test between video-based PBL and paper-based PBL.

In the follow-up survey, we used a chi-square test and a Bonferroni correction method for multiple comparisons to evaluate the recall rate of three groups: video, paper, and non-experienced. It is possible that the period from the conclusion of the PBL tutorials until the follow-up survey could influence recall; thus, we used a Mann-Whitney U test for the video and paper groups regarding the relationship between the presence and absence of recall and the period before the follow-up survey.

## Results

### Target achievement rate of PBL tutorials

The Cronbach’s alpha of the questionnaire was 0.861. Video-based PBL had a significantly higher achievement rate for “The development of an effective clinical reasoning process,” one of the five target items for the university’s PBL tutorials (p=0.042). No significant differences were found between the two groups for the other four items (see Table 1). Video-based PBL was significantly higher in the achievement rates for “imagining the authentic patient (p=0.001)” and “incorporating a comprehensive approach including psychosocial aspects (p<0.001),” as well as “satisfaction with the session (p=0.001)” (see Table 1).

**Table 3:** Results of the follow-up survey about the recall of the experienced cases, Chiba University Hospital in 2015 (N=100)

Group^*^	Presence n (%)	Absence n (%)	c^2^	p value
Video	20 (20.0)	80 (80.0)	24.721	<0.001
Paper	14 (14.0)	86 (86.0)		
Non-experienced	13 (4.3)	287 (95.7)		
Multiple comparison (Bonferroni correction)^†^				
Video	20 (20.0)	80 (80.0)	1.276	0.259
Paper	14 (14.0)	86 (86.0)		
Video	20 (20.0)	80 (80.0)	24.319	<0.001
Non-experienced	13 (4.3)	287 (95.7)		
Paper	14 (14.0)	86 (86.0)	11.134	0.001
Non-experienced	13 (4.3)	287 (95.7)		

*Chi-square test was used to assess these three groups, p<0.05;

†Bonferroni correction for multiple comparison, p<0.0167.

In the comparative survey on which the students preferred video- or paper-based PBL, four answered that they strongly preferred video (3.7%); 46 answered that they preferred video (42.6%); three answered that they strongly preferred paper (2.8%); 21 answered that they preferred paper (19.4%); and 34 answered that there was no difference between the two (31.5%). Many students answered that they preferred video (50 students, 46.3%). The most frequently mentioned reason for preferring video-based PBL was “Ease of imagining the authentic patient.”

### Discussion contents diversity of PBL tutorials

No significant difference was found between video-based and paper-based PBL for the number of ICPC-2 codes, the number of ICPC-2 chapter types, and the rate of P codes (see Table 2).

In the tutor meeting, the opinion was shared that “There were many groups that spent time confirming the video content because it was necessary to extract information from the video in video-based PBL.” Furthermore, the opinion was expressed that “In paper-based PBL the same information is put into words, so no time is required to confirm the contents like in video-based PBL, and the students were able to start discussing the cases immediately.

### Follow-up survey

Significant differences were confirmed (χ^2^=24.721, p<0.001) between the three groups: video, paper, and non-experienced. In multiple comparisons using the Bonferroni correction method, video and paper were significantly higher for recall rate between video and non-experienced (χ^2^=24.319, p<0.001), and between paper and non-experienced (χ^2^=11.134, p=0.001). Although the rate for video tended to be higher than the rate for paper, no significant difference was found between video and paper (see Table 3). For both video and paper, there was no relation between presence or absence of recall and the period before the follow-up survey (see Table 4).

**Table 4:** Comparison between the duration of the follow-up survey and the presence or absence of recall, Chiba University Hospital in 2015 (N=100)

Group	Recall	25^th*^	Median	75^th**^	p value
Video (day)	Presence	265	368	420	0.750
	Absence	250	353	451	
Paper (day)	Presence	209	306	389	0.083
	Absence	265	368	451	

*25th percentile; **75th indicates the 75th percentile.

## Discussion

The Cronbach’s alpha of the questionnaire exceeded 0.8: internal consistency was considered high. This research used simulated videos showing not only medical examinations in examining rooms, but also scenes of the patients' symptoms actually appearing and patients’ daily lives. This should have made imagining the patient easier. As a result, one can guess that it should be possible to take the patient’s psychosocial aspects into consideration, leading to high achievement rates for “imagining the authentic patient” and “incorporating a comprehensive approach.” Video-based PBL had a higher achievement rate for “the development of an effective clinical reasoning process,” as well as higher “satisfaction with the session.” Additionally, in comparisons of the modality of the case, more students preferred video-based PBL. The reason given, echoing research by de Leng B et al^8^ was that it was easy to imagine the authentic patient. Therefore, one would think that video-based PBL is more suitable for learning the actual process of clinical reasoning with consideration of biopsychosocial aspects.

However, no significant difference was found between video-based and paper-based PBL for the discussion contents diversity, as evaluated by the number of ICPC-2 codes, the number of ICPC-2 chapter types, and the rate of P codes. One possible reason is that students felt that video-based PBL made it easy to imagine the patient, and also that they were able to incorporate a comprehensive approach including psychosocial aspects. However, the number of differential diseases was not larger because this image conversely constrained students.

One more factor for no difference in the discussion contents diversity is thought to be the different case recognition processes for the video- and paper-based PBL. That is, video-based PBL required more time for video analysis and problem extraction.^9^ In research by Roy R Basu and colleagues^15^ assessing critical thinking quality, the preferences for the modality of case rate and evaluations of learning effect were higher for video-based PBL than paper-based PBL for both students and tutors. However, video-based PBL involved less deep thinking. The reason pointed out is that thinking is impacted by the large amount of information obtained from videos, as well as the time required to process it. In research by LA Woodham et al^12^ students recognized the benefit of video-based PBL, such as the sense that they had experienced clinical reasoning like physicians and the ability to obtain visual information, but many students still preferred paper-based to video-based PBL. This is because video-based PBL requires advanced abilities to select the necessary information from the video and make judgments, which requires time. This suggests that video-based PBL may hinder PBL progression and students’

clinical reasoning. In this research as well, the view was raised that time was required in the video-based PBL for group members to discuss the video contents and its analyses. Paper-based PBL provided the same information put into words, so this time was not necessary.

However, the focus group discussion clarified that a great deal of information could be obtained from video-based PBL, resulting in the presence of recall.^8^ In the follow-up survey for this research, based on the hypothesis that it would be easy for the target students to recall the cases experienced in the PBL tutorials when looking at disease names, a quantitative evaluation was conducted of recall rate for the video and paper groups. This evaluation found that both video and paper had more significant recall of the chief complaints than lack of experience, confirming that video-based PBL has learning effects similar to those of traditional paper-based PBL. No significant difference was found between video and paper. However, a higher percentage of students could recall the chief complaints about video than from paper, which suggests that video-based PBL is more memorable, a result that accords with previous research. It is possible that a beta error is the reason that no significant difference was found, so the sample size must be enlarged to reconsider this question.

### Limitation

In the evaluation of discussion contents, only differential disease names were extracted from the PBL tutorial audio recordings. For instance, “depression” was counted, but “psychogenetic” was not counted, so even when clinical conditions were discussed, they were not counted if no specific diseases were named. For both groups, there was a low rate of psychogenic diseases among the differential diseases. We thought that the students were less well versed in psychogenic diseases than in biological diseases, which might indicate that psychogenic discussion is undervalued.

## Conclusions

When using patient-simulated videos that included patients’ daily lives, the rate of psychosocial issues during the discussion was not higher, due to factors such as the impacts of being constrained by the video images. However, video made imagining the authentic patient easier and also made it easier to realize a comprehensive approach including psychosocial aspects, which is required for actual clinical practice. In the follow-up survey, there was a tendency for patient-simulated videos to inspire better recall than paper mediums.

Patient-simulated video with patients’ daily life facilitates consideration that includes psychosocial aspects and is a more practical clinical method in PBL. In the future, we would like to create patient-simulated videos for each symptom and to undertake large-scale surveys of the outcome assessments of students and changes in the burdens on the tutors.

### Acknowledgements

This study was supported by JSPS Grant-in-Aid for Challenging Exploratory Research Grant Number 25670242, and Initiative for Realizing Diversity in the Research Environment.

### Conflict of Interest

The authors declare that they have no conflict of interest.

## References

[1] Neufeld VR, Barrows HS (1974). The "McMaster Philosophy": an approach to medical
education.. J Med Educ.

[2] Davis MH (1999). AMEE Medical Education Guide No. 15: Problem-based learning: a practical
guide.. Med Teach.

[3] Adler RH (2009). Engel's biopsychosocial model is still relevant today.. J Psychosom Res.

[4] Spector A, Orrell M (2010). Using a biopsychosocial model of dementia as a tool to guide clinical
practice.. Int Psychogeriatr.

[5] Azer SA (2007). Twelve tips for creating trigger images for problem-based learning
cases.. Med Teach.

[6] Dong C, Goh PS (2015). Twelve tips for the effective use of videos in medical
education.. Med Teach.

[7] Dequeker J, Jaspaert R (1998). Teaching problem-solving and clinical reasoning: 20 years experience with
video-supported small-group learning.. Med Educ.

[8] de Leng B, Dolmans D, van de Wiel M, Muijtjens A, van der Vleuten C (2007). How video cases should be used as authentic stimuli in problem-based
medical education.. Med Educ.

[9] Balslev T, de Grave WS, Muijtjens AM, Scherpbier AJ (2005). Comparison of text and video cases in a postgraduate problem-based learning
format.. Med Educ.

[10] Chan LK, Patil NG, Chen JY, Lam JC, Lau CS, Ip MS (2010). Advantages of video trigger in problem-based learning.. Med Teach.

[11] Ghanchi NK, Khan S, Afridi A, Sajid S, Afzal S, Ahmed I, Ahmed R, Ghias K (2013). Video or paper for delivery of problem-based learning
cases?. Med Educ.

[12] Woodham LA, Ellaway RH, Round J, Vaughan S, Poulton T, Zary N (2015). Medical Student and Tutor Perceptions of Video Versus Text in an
Interactive Online Virtual Patient for Problem-Based Learning: A Pilot
Study.. J Med Internet Res.

[13] WONCA International Classification Committee. ICPC-2: International classification of primary care. 2nd ed. Oxford: Oxford Medical Publication; 1998.

[14] Norwegian Centre for Informatics in Health and Social Care. Norway: International Classification of Primary Care. 2nd ed. Version 5.0 [cited 19 May 2015]; Available from: http://www.kith.no/templates/kith_WebPage____1111.aspx.

[15] Basu Roy R, McMahon GT (2012). Video-based cases disrupt deep critical thinking in problem-based
learning.. Med Educ.

